# Supplementary material for: Sodium and Potassium Intakes and Cardiovascular Risk Profiles in Childhood Cancer Survivors: The SCCSS-Nutrition Study
Source: Nutrients. 2019 Dec 24;12(1):57. doi: 10.3390/nu12010057 (PMC7019213; doi:10.3390/nu12010057)
Supplement: Supplementary file 1 [file nutrients-12-00057-s001.pdf]

Supplementary material

Supplementary Table S1. Scoring method and mean scores (95%CI) of components of the modified Alternate Healthy Eating Index[1,2] of adult childhood cancer survivors (CCSs) who filled in the food frequency questionnaire by sex

Components	Criteria for minimum score (0)	Criteria for maximum score (10)	Score in CCSs[13] mean (95%CI)	
			male (*n* =401)	female (*n* =401)
Vegetables, excluding potatoes (servings/day)[3]	0	≥ 5	4.6 (4.4; 4.9)	5.5 (5.3; 5.7)
Fruit, excluding juice (servings/day)[4]	0	≥ 4	2.9 (2.6; 3.2)	3.9 (3.6; 4.2)
Whole grains (g/day)[5]				
Males	0	≥ 90	3.3 (3.0; 3.7)	
Females	0	≥ 75		4.6 (4.3; 5.0)
Sugar-sweetened beverage and fruit juice (servings/day)[6]	≥ 1	0	3.8 (3.5; 4.2)	5.0 (4.6; 5.3)
Nuts, seeds, legumes, and tofu (servings/day)[7]	0	≥ 1	1.9 (1.6; 2.2)	2.5 (2.2; 2.8)
Red and processed meat (servings/day)[8]	≥ 1.5	0	3.5 (3.2; 3.8)	5.7 (5.4; 6.0)
Trans fat (% of total energy intake)[9]	≥ 4	≤ 0.5	9.4 (9.4; 9.4)	9.4 (9.3; 9.4)
Fish, excluding processed products (g/day)	0	32.4	5.2 (4.8; 5.6)	5.0 (4.6; 5.3)
PUFA (% of total energy intake)[10]	≤2	≥10	4.8 (4.6; 5.1)	5.1 (4.8; 5.3)
Sodium[11], mg/day	Highest decile	Lowest decile	5.3 (5.0; 5.6)	5.3 (5.0; 5.6)
Alcohol (drinks/day)[12]				
Males	≥ 3.5	0.5 - 2.0	5.4 (5.0; 5.7)	
Females	≥ 2.5	0.5 - 1.5		4.0 (3.7; 4.3)
**Total**	**0**	**100**	**50.3 (49.1; 51.5)**	**56.0 (54.8; 57.2)**

CCSs, childhood cancer survivors; CI, confidence interval, ICCC-3, International Childhood Cancer Classification, 3rd edition; PUFA, polyunsaturated fatty acids
[1] Adapted from Chiuve et al. J Nutr 2012 142(6):1009-18.
[2] Intermediate food intake was scored proportionately between the minimum score 0 and the maximum score 10.
[3] One serving was equal to 118.3 g of raw or cooked vegetables or 250 g of vegetable soup. Dried vegetables were not included.
[4] One serving was equal to 118.3 g of raw or cooked fruit. Dried fruit was not included.
[5] Whole grain products like whole grain bread and muesli were included.
[6] One serving was equal to 226.8 g.
[7] One serving was equal to 28.4 g.
[8] One serving was equal to 113.4 g of red meat or 42.5 g of processed meat.
[9] Consumption was estimated based on the assumption that each food contains a maximum of 2 g of trans fat per 100 g of total fat, as defined in Swiss regulation.
[10] The highest score was given to individuals with ≥10% of total energy intake from PUFA.
[11] Values in lowest decile were ≤1084 mg/d of sodium in females and ≤1168 mg/d in males and in highest decile were ≥2802 mg/d in females and ≥2969 mg/d in males.
[12] One drink was 113.4 g of wine, 340.2 g of beer or 42.5 g of liquor. A score of 2.5 was given to nondrinkers.
[13] Adjusted for age at survey and ICCC-3 diagnosis.

Supplementary Table S2. Sociodemographic and lifestyle characteristics of adult childhood cancer survivors by participants and nonparticipants to the SCCSS-Nutrition study

	FFQ			Morning fasting spot urine		
	**Participants (*n* = 802)**	**Non-participants[1] (*n* = 797)**	***p-value*[2]**	**Participants (*n* = 111)**	**Non-participants[3] (*n* = 110)**	***p-value*[2]**
Characteristics *n (%)*						
**Sex**						
Females	401 (50)	354 (44)	***0.025***	62 (56)	57 (52)	***0.547***
Males	401 (50)	443 (56)		49 (44)	53 (48)	
**Age at follow-up survey**[4], median (IQR)	34.6 (28.8; 41.1)	32.0 (27.1; 38.7)	***0.520***	39.1 (30.7; 45.2)	33.4 (26.9; 40.4)	***0.290***
≤30 y	248 (31)	328 (41)[d]	***<0.001***	26 (23)	44 (40)	***0.010***
31-39 y	320 (40)	305 (38)		37 (33)	37 (34)	
≥40 y	234 (29)	164 (21)		48 (43)	29 (26)	
**Country of birth**						
Switzerland	763 (95)	737 (92)	***0.027***	101 (91)	98 (89)	***0.637***
Other	39 (5)	60 (8)		10 (9)	12 (11)	
**Language region within Switzerland**						
German speaking	570 (71)	581 (73)	***0.548***	-	-	***NA***
French speaking	214 (27)	195 (24)		111 (100)	110 (100)	
Italian speaking	18 (2)	21 (3)		-	-	
**Education (highest degree)**[5]						
Lower than university	686 (86)	699 (88)	***0.203***	69 (62)	71 (65)	***0.713***
University	116 (14)	98 (12)		42 (38)	39 (35)	
**Living situation**[5]						
Alone	131 (16)	129 (16)	***0.936***	19 (17)	24 (22)	***0.377***
With others	671 (84)	668 (84)		92 (83)	86 (78)	
**Physical activity**[5,6]						
Inactive	157 (20)	204 (26)	***0.004***	32 (29)	32 (29)	***0.966***
Active	645 (80)	593 (74)		79 (72)	78 (71)	
**Smoking status**[5]						
Current	151 (19)	207 (26)	***0.002***	25 (23)	21 (19)	***0.261***
Former	97 (12)	79 (10)		15 (14)	24 (22)	
Never	554 (69)	511 (64)		71 (64)	65 (59)	
**BMI at baseline survey**[5]						
Obese, ≥30 kg/m²	46 (6)	59 (7)	***0.548***	10 (9)	14 (13)	***0.134***
Overweight, ≥25 to <30 kg/m²	146 (18)	141 (18)		15 (14)	25 (23)	
Normal, ≥18.5 to <25 kg/m²	546 (68)	540 (68)		79 (71)	68 (62)	
Underweight, <18.5 kg/m²	64 (8)	57 (7)		7 (6)	3 (3)	

BMI, body mass index; CCSs, childhood cancer survivors; FFQ, food frequency questionnaire; IQR, interquartile range; NA, not applicable; SCCSS, Swiss Childhood Cancer Survivor Study

[1] This includes 635 CCSs who did not respond, 45 who refused, 11 who were pregnant or breastfeeding, 35 with missing dietary data, and 71 with unreliable dietary data.

[2] p-value calculated from chi-square statistics (categorical, 2-sided test) or Kruskal-Wallis test (continuous, nonparametric) to compare differences between participants to nonparticipants.

[3] This includes 9 CCSs with no valid address available anymore or being abroad, 15 who refused, and 86 who did not respond.

[4] Age at follow-up survey is calculated for FFQ nonparticipants by taken the average participants' date of filling in the questionnaire.

[5] Based on information from the SCCSS baseline questionnaire filled in between 2007-2013.

[6] Active: ≥150 minutes of moderate intense or 75 minutes of vigorous intense or a combination of moderate and vigorous intense physical activity per week.

Supplementary Table S3. Clinical characteristics of adult childhood cancer survivors by participants and nonparticipants to the SCCSS-Nutrition study

	FFQ			Morning fasting spot urine		
Characteristics, *n (%)*	**Participants (*n* = 802)**	**Non-participants[1] (*n* = 797)**	***p-value*[2]**	**Participants (*n* = 111)**	**Non-participants[3] (*n* = 110)**	***p-value*[2]**
**ICCC-3 diagnosis**						
I: Leukemia	246 (31)	264 (33)	***<0.001***	30 (27)	27 (25)	***0.607***
II: Lymphoma	173 (22)	139 (17)		30 (27)	30 (27)	
III: CNS tumor	81 (10)	140 (18)		9 (8)	17 (15)	
IV: Neuroblastoma	28 (3)	31 (4)		4 (4)	3 (3)	
V: Retinoblastoma	12 (2)	22 (3)		2 (2)	3 (3)	
VI: Renal tumor	52 (6)	41 (5)		4 (4)	3 (3)	
VII: Hepatic tumor	6 (<1)	3 (<1)		1 (<1)	1 (<1)	
VIII: Bone tumor	50 (6)	29 (4)		11 (10)	5 (5)	
IX: Soft tissue sarcoma	66 (8)	32 (4)		7 (6)	10 (9)	
X: Germ cell tumor	43 (5)	42 (5)		8 (7)	4 (4)	
XI & XII: Other tumor	26 (3)	17 (2)		4 (4)	3 (3)	
Langerhans cell histiocytosis	19 (2)	37 (5)		1 (<1)	4 (4)	
**Age at diagnosis**,[4] years	9.7 (3.9; 13.9)	8.0 (3.8; 13.1)	***0.010***	11.5 (5.0; 15.6)	10.7 (4.6; 15.8)	***0.696***
**Time since diagnosis**,[4] years	26.1 (20.2; 31.7)	24.0 (19.5; 30.3)	***0.186***	28.3 (21.0; 32.7)	22.8 (18.5; 30.1)	***0.004***
**Any irradiation**	292 (36)	293 (37)	***0.883***	40 (36)	37 (34)	***0.708***
Chest irradiation	87 (11)	67 (8)	***0.098***	15 (14)	10 (9)	***0.299***
<30 Gy	31 (4)	21 (3)	***0.218***	3 (3)	3 (3)	***0.498***
≥30 Gy	56 (7)	46 (6)		12 (11)	7 (6)	
**Any chemotherapy**	642 (80)	606 (76)	***0.052***	80 (72)	78 (71)	***0.848***
Anthracyclines	298 (37)	298 (37)	***0.923***	39 (35)	26 (24)	***0.061***
**HSCT**	30 (4)	34 (4)	***0.592***	5 (5)	7 (6)	***0.542***
**History of relapse**	99 (12)	107 (13)	***0.519***	18 (16)	15 (14)	***0.591***

CCSs, childhood cancer survivor; CNS, central nervous system; Gy, gray; HSCT, hematopoietic stem cell transplantation; ICCC-3, International Childhood Cancer Classification, 3rd edition; SCCSS, Swiss Childhood Cancer Survivor Study

[1] This includes 635 CCSs who did not respond, 45 who refused, 11 who were pregnant or breastfeeding, 35 with missing dietary data, and 71 with unreliable dietary data.

[2] p-value calculated from two-sample mean comparison test (t test) or chi-square statistics comparing participants excluded from analyses, nonparticipants, and non-eligible CCS to participants (2-sided test).

[3] This includes 9 CCSs with no valid address available anymore or being abroad, 15 who refused, and 86 who did not respond.

[4] Values are medians with interquartile ranges (IQRs).

Supplementary Table S4. Dietary intake based on self-reported food frequency questionnaire (FFQ) data of adult childhood cancer survivors by participants to the SCCSS-Nutrition study

	DACH recommendations[1]		FFQ participants (n = 802)		FFQ but not spot urine participants n = 691		Spot urine participants (n = 111)	
	**male**	**female**	**mean ± SD**	**% DACH[2]**	**mean ± SD**	**% DACH[2]**	**mean ± SD**	**% DACH[2]**
Total energy, kcal[3]	2300-3400	1800-2600	1639 ± 581	65	1654 ± 595	65	1543 ± 481	62
**Macronutrients**								
Protein, g	57	48	66.0 ± 29.3	126	67.0 ± 30.3	127	60.1 ± 21.2	116
% of energy[4]	10-20	10-20	16.1 ± 3.6	107	16.2 ± 3.6	108	15.7 ± 3.2	105
Vegetal, g			18.2 ± 7.6		18.2 ± 7.8		17.7 ± 6.6	
Animal, g			47.9 ± 26.6		48.8 ± 27.6		42.4 ± 19.1	
Carbohydrates, g[5]	225-275	225-275	181 ± 74	72	182 ± 76	73	175 ± 65	70
% of energy[4, 5]	45-55	45-55	44.1 ± 8.9	88	44.0 ± 9.0	88	45.1 ± 8.0	90
Mono/disaccharides, g			86 ± 47		86 ± 47		86 ± 48	
Polysaccharides, g			94 ± 44		95 ± 46		88 ± 36	
Total fiber, g[5]	≥30	≥30	12.2 ± 6.5	41	12.2 ± 6.5	41	12.1 ± 6.3	40
Total fat, g			67.9 ± 28.0		68.8 ± 28.4		62.7 ± 24.2	
Total fat, % of energy[4]	30-40	30-40	37.3 ± 6.9	107	37.4 ± 7.0	107	36.4 ± 6.5	104
Saturated fat, g			25.1 ± 11.6		25.5 ± 11.6		23.2 ± 11.0	
Saturated fat, % of energy[6]	<10	<10	13.7 ± 3.2	137	13.7 ± 3.1	137	13.3 ± 3.4	133
Monounsaturated fat, g			28.4 ± 12.6		28.8 ± 12.9		26.1 ± 10.4	
Polyunsaturated fat, g			8.6 ± 3.7		8.7 ± 3.8		7.9 ± 3.4	
Cholesterol, mg[4]	300	300	341 ± 206	114	349 ± 215	116	288 ± 131	96
**Micronutrients**								
Vitamins								
A, mg-RE[7]	1.0	0.8	0.7 ± 0.7	78	0.7 ± 0.7	78	0.7 ± 0.4	79
D, µg	20	20	2.4 ± 2.0	12	2.4 ± 2.0	12	2.4 ± 1.8	12
Minerals								
Calcium (Ca), mg	1000	1000	889 ± 467	89	901 ± 474	90	810 ± 416	81
Iron (Fe), mg	10[4]	15	9.0 ± 3.5	72	9.1 ± 3.7	73	8.4 ± 2.7	66
Phosphorus (P), mg	700	700	1227 ±448	175	1248 ± 462	178	1093 ± 320	156
Potassium (K), mg	4000	4000	2740 ± 1072	68	2759 ± 1080	68	2618 ± 1019	65
Sodium (Na), mg[8]	1500	1500	2844 ± 659	190	2859 ± 656	190	2747 ± 674	183
Alcohol, g[9]	20	10	5.7 ± 7.7	38	5.7 ± 8.0	38	5.7 ± 6.0	40

DACH, dietary recommendations for Germany (D), Austria (A) and Switzerland (CH); FFQ, food frequency questionnaire; SCCSS, Swiss Childhood Cancer Survivor Study; SD, standard deviation

[1] DACH recommendations for the general population age 20-50 years, excluding pregnant and lactating females, 2015.

[2] Percentage of mean intake in relation to the DACH recommended intake level * 100. Recommended intake is estimated on the basis of the age-sex groups of the DACH guidelines, weighted by the age and sex distribution of the study population. For alcohol intake the maximum tolerated dosage was taken.
[3] Depending on physical activity level (PAL, 1.4-1.8) and age.
[4] Federal Food Safety and Veterinary Office FSVO, www.naehrwertdaten.ch.
[5] BLV (2009). Kohlenhydrate in unserer Ernährung – Empfehlungen des BLV. www.blv.admin.ch.
[6] BLV (2012). Fett in unserer Ernährung – Empfehlung des BLV. www.blv.admin.chv, recommendations as a percentage of the daily energy demand and based on a daily energy intake of 2000 kcal.
[7] Total vitamin A = retinol + carotene/12, expressed in µg RAE.
[8] Na intake based on FFQ data was calculated with the equation: (8.20 + 0.38*FFQ in males)/2.54 and (4.55 + 0.67*FFQ in females)/2.54, with 1 g Na = 2.54 g NaCl see Ref 35. Intake based on FFQ data only gives a mean daily intake of 1.8 g ± 0.7 for females and 1.9 g ± 0.8 for males.
[9] Maximum tolerated dosage.

Supplementary Table S5. Sodium (Na) and potassium (K) intake based on food frequency questionnaire (FFQ) data of adult childhood cancer survivors (CCSs) by language region within Switzerland

		CCSs									
	DACH[1]	FFQ participants *n* = 802						Both FFQ and spot urine participants *n* = 111		FFQ but not spot urine participants *n* = 691	
		Total *n* = 802		German speaking *n* = 570		French/Italian speaking *n* = 232					
		mean ± SD	% DACH[2]	mean ± SD	% DACH[2]	mean ± SD	% DACH[2]	mean ± SD	% DACH[2]	mean ± SD	% DACH[2]
Na[3], g	1.5	2.8 ± 0.7	190	2.9 ± 0.7	190	2.8 ± 0.7	188	2.7 ± 0.7	183	2.9 ± 0.7	191
K, g	4.0	2.7 ± 1.1	68	2.8 ± 1.1	69	2.6 ± 1.1	66	2.6 ± 1.0	65	2.8 ± 1.1	69
Na:K ratio[4]	-	*1.2 ± 0.5*	-	*1.2 ± 0.5*	-	*1.2 ± 0.6*	-	*1.2 ± 0.5*	-	*1.2 ± 0.5*	-

CCS, childhood cancer survivors, DACH, dietary recommendations for Germany (D), Austria (A) and Switzerland (CH); FFQ, food frequency questionnaire, Na, sodium; K, potassium; SD, standard deviation

[1] DACH recommendations for the general population age 20-50 years, excluding pregnant and lactating females, 2015. Recommendations for Na and K are similar for males and females.

[2] Percentage of mean intake in relation to the DACH recommended intake level * 100. Recommended intake is estimated on the basis of the age-sex groups of the DACH guidelines, weighted by the age and sex distribution of the study population. For alcohol intake the maximum tolerated dosage was taken.

[3] Na intake based on FFQ data was calculated with the equation: (8.20 + 0.38*FFQ in males)/2.54 and (4.55 + 0.67*FFQ in females)/2.54, with 1 g Na = 2.54 g NaCl see Ref 35. Intake based on FFQ data only gives a mean daily intake of 1.8 g ± 0.7 for females and 1.9 g ± 0.8 for males.

[4] Na:K ratio was calculated by dividing the sodium intake (g/day) by the potassium intake (g/day).

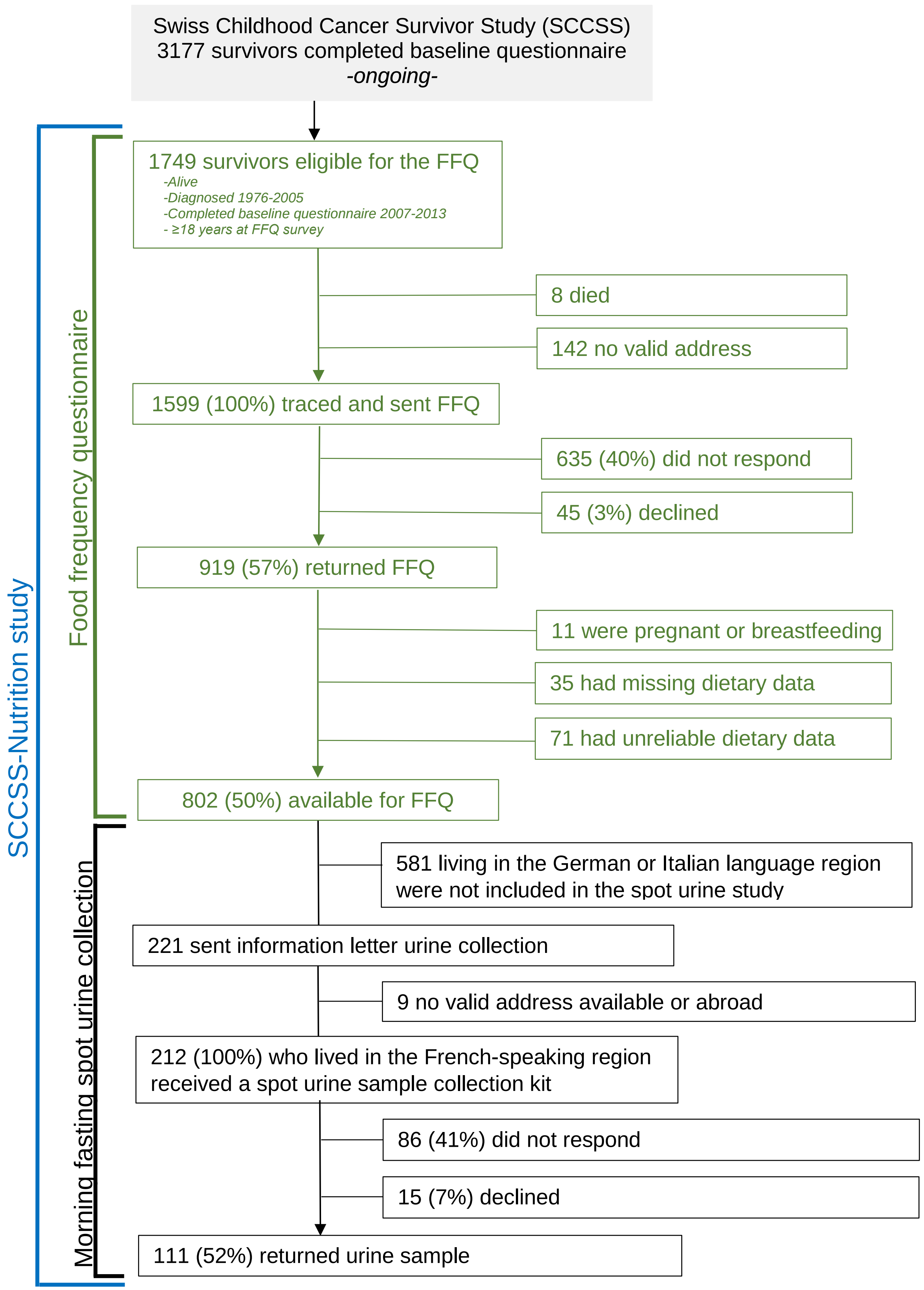

Figure 1. Response rates in the Swiss Childhood Cancer Survivor Study (SCCSS)-Nutrition study

FFQ, food frequency questionnaire

24. **Système cardiovasculaire**

	Déjà eu?		Quand?	Encore présent?	
	**Non**	**Oui**		**Non**	**Oui**
Pression artérielle élevée (hypertension à répétition)	☐	☐	____ (année)	☐	☐
Irrégularité du rythme cardiaque ou palpitations (arythmie), qui a-ont nécessité une investigation cardiologique	☐	☐	____ (année)	☐	☐
Insuffisance cardiaque (insuffisance du muscle cardiaque)	☐	☐	____ (année)	☐	☐
Maladie coronarienne (angine de poitrine: sensation de pression accompagné de douleurs au niveau de la poitrine à cause d'une moindre oxygénation du cœur)	☐	☐	____ (année)	☐	☐
Infarctus du myocarde (crise cardiaque)	☐	☐	____ (année)	☐	☐
Thrombose veineuse profonde (p. ex. thrombose d'une jambe)	☐	☐	____ (année)	☐	☐
Embolie pulmonaire	☐	☐	____ (année)	☐	☐
Attaque d'apoplexie (accident cérébrovasculaire etc.)	☐	☐	____ (année)	☐	☐
Artériosclérose (Durcissement des artères des jambes, de la carotide ou des artères coronaires)	☐	☐	____ (année)	☐	☐
Autres problèmes concernant le système cardiovasculaire **Si oui**, veuillez préciser ____________________ ____________________	☐	☐	____ (année)	☐	☐

Avez-vous eu des opérations du cœur ou du système cardiovasculaire depuis 2007?
Si oui, veuillez indiquer toutes les opérations à la page 9, question 18.

Supplementary Figure 2A. Self-reported cardiovascular related medical history questions in French assessed within the Swiss Childhood Cancer Survivor Study (SCCSS)-Nutrition Study

24. Herz- und Kreislaufsystem

	Irgendwann im Leben? Nein	Irgendwann im Leben? Ja	Seit wann?	Aktuell noch vorhanden? Nein	Aktuell noch vorhanden? Ja
Hoher Blutdruck (arterielle Hypertonie, wiederholt gemessen)	☐	☐	____ (Jahr)	☐	☐
Herzrhythmusstörungen (Arrhythmien: unregelmässige Herzschläge), welche von einem Kardiologen (Herzspezialisten) abgeklärt werden mussten	☐	☐	____ (Jahr)	☐	☐
Herzinsuffizienz (Herzmuskelschwäche)	☐	☐	____ (Jahr)	☐	☐
Koronare Herzkrankheit (Angina pectoris: Engegefühl mit Schmerzen in der Brust, das durch einen Sauerstoffmangel im Herzen auftritt)	☐	☐	____ (Jahr)	☐	☐
Herzinfarkt	☐	☐	____ (Jahr)	☐	☐
Tiefe Venenthrombose (Blutgerinnsel z.B. im Bein)	☐	☐	(Jahr) ____	☐	☐
Lungenembolie	☐	☐	____ (Jahr)	☐	☐
Schlaganfall (Hirninfarkt oder Hinrblutung)	☐	☐	____ (Jahr)	☐	☐
Arteriosklerose (Arterienverkalkung der Beine, Halsschlagader oder Herzkranzgefässe)	☐	☐	____ (Jahr)	☐	☐
Andere Probleme, die das Herz- und Kreislaufsystem betreffen.	☐	☐	____ (Jahr)	☐	☐

Falls Ja, bitte beschreiben Sie die Probleme näher:

____

____

Wurden Sie seit 2007 am Herzen operiert oder hatten Sie Eingriffe am Herz- oder Kreislaufsystem? Falls Ja, bitte geben Sie alle Operationen auf Seite 9, Frage 18 an.

Supplementary Figure 2B. Self-reported cardiovascular related medical history questions in German assessed within the Swiss Childhood Cancer Survivor Study (SCCSS)-Nutrition Study
